# Personalized Predictive Hemodynamic Management for Gynecologic Oncologic Surgery: Feasibility of Cost–Benefit Derivatives of Digital Medical Devices

**DOI:** 10.3390/jpm14010058

**Published:** 2023-12-30

**Authors:** Luciano Frassanito, Rossella Di Bidino, Francesco Vassalli, Kristian Michnacs, Pietro Paolo Giuri, Bruno Antonio Zanfini, Stefano Catarci, Nicoletta Filetici, Chiara Sonnino, Americo Cicchetti, Giovanni Arcuri, Gaetano Draisci

**Affiliations:** 1 Department of Emergency, Anesthesiologic and Intensive Care Sciences, IRCCS Fondazione Policlinico A. Gemelli, Largo A. Gemelli 8, 00168 Rome, Italy; pietropaolo.giuri@policlinicogemelli.it (P.P.G.); brunoantonio.zanfini@policlinicogemelli.it (B.A.Z.); stefano.catarci@policlinicogemelli.it (S.C.); nicoletta.filetici@policlinicogemelli.it (N.F.); chiara.sonnino@policlinicogemelli.it (C.S.); gaetano.draisci@policlinicogemelli.it (G.D.); 2 Department of Health Technology, IRCCS Fondazione Policlinico A. Gemelli, 00168 Rome, Italy; rossella.dibidino@policlinicogemelli.it (R.D.B.); giovanni.arcuri@policlinicogemelli.it (G.A.); 3 Department of Critical Care and Perinatal Medicine, IRCCS Istituto G. Gaslini, 16147 Genoa, Italy; francescovasssalli@gmail.com; 4 Edwards Lifescience GmbH, 85748 München, Germany; kristian_michnacs@edwards.com; 5 Department of Management Studies, Faculty of Economics, Catholic University of Sacred Heart, 00168 Rome, Italy; americo.cicchetti@unicatt.it

**Keywords:** intraoperative hypotension, machine learning, healthcare resource utilization, gynecologic oncologic surgery, length of stay

## Abstract

Background: Intraoperative hypotension is associated with increased perioperative complications, hospital length of stay (LOS) and healthcare expenditure in gynecologic surgery. We tested the hypothesis that the adoption of a machine learning-based warning algorithm (hypotension prediction index—HPI) might yield an economic advantage, with a reduction in adverse outcomes that outweighs the costs for its implementation as a medical device. Methods: A retrospective-matched cohort cost–benefit Italian study in gynecologic surgery was conducted. Sixty-six female patients treated with standard goal-directed therapy (GDT) were matched in a 2:1 ratio with thirty-three patients treated with HPI based on ASA status, diagnosis, procedure, surgical duration and age. Results: The most relevant contributor to medical costs was operating room occupation (46%), followed by hospital stay (30%) and medical devices (15%). Patients in the HPI group had EURO 300 greater outlay for medical devices without major differences in total costs (GDT 5425 (3505, 8127), HPI 5227 (4201, 7023) *p* = 0.697). A pre-specified subgroup analysis of 50% of patients undergoing laparotomic surgery showed similar medical device costs and total costs, with a non-significant saving of EUR 1000 in the HPI group (GDT 8005 (5961, 9679), HPI 7023 (5227, 11,438), *p* = 0.945). The hospital LOS and intensive care unit stay were similar in the cohorts and subgroups. Conclusions: Implementation of HPI is associated with a scenario of cost neutrality, with possible economic advantage in high-risk settings.

## 1. Introduction

Intraoperative hypotension (IOH) represents a common complication during general anesthesia, with a reported incidence that varies with the chosen threshold between 5 and 99% [1]. IOH, defined as mean arterial blood pressure (MAP) < 65 mm Hg for at least 1 min [1], is associated with adverse perioperative outcomes, including cardiovascular events [2,3], acute kidney injury [2,3,4] and increased mortality [5,6,7]. It is now well recognized that organ damage is proportional to the number, duration and severity of hypotensive events: even a short period of profound IOH can be harmful to patients [8]. 

Therefore, the relationship between IOH and organ disfunction is dose-dependent, with no apparent safe period of hypotension [9]. Given the potentially avoidable nature of the hazard and the extent of the exposed population, several authors consider IOH to be a serious public health issue that should not be ignored in any age group [8,9]. Hence, strict blood pressure control and hemodynamic optimization is warranted throughout the perioperative period, and advanced invasive and non-invasive hemodynamic monitoring should become a new standard of care in this setting [10].

Traditional hemodynamic management is reactive, which involves taking remedial actions when the MAP drops under 65 mm Hg. With a “pro-active” approach, instead, the treatment is administered even before IOH manifests, possibly avoiding any hypotensive event. Therefore, the capability to predict impending IOH becomes crucial. Predicting the risk of a dynamic process of multi-state transition from a state of covert hemodynamic instability to frank hypotension is made possible using artificial intelligence [11,12]. Machine learning is a subset of artificial intelligence; this science enables systems to automatically learn from data and improve from experience without being explicitly programmed, capturing and analyzing a vast number of different variables from large data sets, the so called “model features”, in order to develop predictive models [13]. Machine learning has growing applications in healthcare [13].

Several attempts to use algorithms as an aid in anesthesiology practice recently received renewed attention, with the aim of optimizing patients’ perioperative status; they are primarily focused on detecting early hemodynamic instability and predicting hypotension [11,12]. 

In this regard, the hypotension prediction index (HPI), a machine learning-derived algorithm developed by Edwards Lifesciences, provides a real-time analysis of high-fidelity arterial waveforms and estimates the patient’s likelihood of developing a hypotensive event in the near future [14,15,16]. The HPI technology can also be activated in minimally invasive monitoring mode [17]. When using an Acumen IQ sensor connected to a radial arterial catheter, it provides the clinician with continuous information regarding the likelihood of a patient experiencing a hypotensive event and the associated hemodynamic consequences. The accuracy of the presented measurements is based upon several factors: quality of the arterial pressure waveform (i.e., not damped), proper zeroing and alignment of the arterial pressure sensor, and accurate notation of patient demographics (age, gender, height and weight) [18]. The HPI has been validated on surgical patients with a high sensitivity and specificity in predicting hypotension 5, 10 and 15 min before the event [14,15]. The HPI algorithm evaluates the arterial waveform from the Acumen IQ sensor and updates advanced hemodynamic variables, including HPI, every 20 s [14]. The HPI software displays a dimensionless value ranging from 0 to 100, with higher values indicating a higher likelihood of a hypotensive event. If the HPI value exceeds 85 for two consecutive 20-second updates or reaches 100 at any time, a high alert pop-up window will appear, prompting the anesthesiologist or nurse to review the patient hemodynamics using the Acumen HPI software secondary monitor screen. 

The use of cardiac output monitoring to guide administration of intravenous fluid and inotropic drugs as part of a hemodynamic therapy algorithm has been shown to improve tissue perfusion and oxygenation [19]. The so-called goal-directed hemodynamic therapy (GDT) refers to the use of a protocol to optimize cardiac output-based hemodynamic targets, and to the treatments used to reach these targets [19]. Recent evidence shows that the application of HPI-based hemodynamic guidance in combination with a GDT allows for a reduction in IOH compared to the current standard of care or to a GDT alone [20,21].

Gynecologic oncologic surgery for cancer mass reduction is carried out through laparoscopy or laparotomy, and is often associated with unstable hemodynamics and significant blood loss [21,22]. Hypotension during oncologic surgery is common, and since it is associated with the potential for harm, it requires prompt evaluation and treatment [21]. Extensive fluid resuscitation in peritoneal cancer patients is associated with a poor postoperative outcome, and avoiding fluid overload is recommended [22]. On the other hand, avoiding low blood pressure preserves organ perfusion [3].

The impact of this strategy on difficult perioperative outcomes is still uncertain, due to underpowered trials to investigate such rare occurrences [23]. Unfortunately, the nature of economic evidence about hospital costs and healthcare utilization is still very heterogeneous. North American economic data showed that one minute in the gynecologic operating room costs between USD 46 [24] up to USD 76 [25], while the price for a single day of hospitalization varied between EUR 230 in general hospitals and EUR 323 in university hospitals [26] in the Netherlands, with a three- to four-fold increase in case of intensive care unit (ICU) admission in mean per day in Belgium hospital cost evaluations [27]. 

The potential economic impact of the HPI software is also relevant to understand. According to a recent Spanish study, the application of HPI technology in non-cardiac surgery reduced hospital length of stay (LOS) by 2 days [28]. Despite the economic advantage of reduced LOS, it must be weighed against the cost impact for the implementation of HPI as a technical element of the operating room and ICU hospital infrastructure. The primary goal of this study was to retrospectively analyze real-world data of hospital costs related to the adoption of HPI software and Acumen IQ sensor technology in the clinical practice of our hospital.

## 2. Materials and Methods

This is a retrospective cost–benefit study conducted at the IRCCS Policlinico Universitario Agostino Gemelli Foundation in Rome and approved by the Internal Ethic Committee (protocol number 0022457/22, protocol ID 4902) on 30 June 2022.

The study included 99 adult female patients undergoing elective gynecologic oncologic surgery between January 2020 and July 2021. Thirty-three patients receiving hemodynamic guidance and monitoring with HPI software and Acumen IQ sensor technology (HPI group) were matched with sixty-six patients treated according to standard GDT (control group). Matching occurred in a 2:1 ratio based on ASA physical status, diagnosis at hospital admission, type of procedure, duration of surgery and patient age. 

Institutional anesthetic management for major gynecologic oncologic surgery is standardized, and it was the same in the two groups. 

All of the patients were offered central neuraxial anesthesia for postoperative pain management, either 100 μg of intrathecal morphine for expected laparoscopic surgery or T12-L1 epidural catheter placement for expected laparotomic surgery. 

General anesthesia induction was performed with 2–3 mg·kg^−1^ of propofol, 0.2 mcg·kg^−1^ of sufentanil and 0.6 mg·kg^−1^ of rocuronium bromide. Sevoflurane was used for maintenance, to achieve a target bispectral index value between 40 and 50. Intravenous sufentanil was supplemented between 0.1 and 0.2 mcg·kg^−1^·h^−1^. Mechanical ventilation was performed with a tidal volume of 8 mL·kg^−1^ of predicted body weight, with a positive end-expiratory pressure of 5 cmH_2_O, and an inspired oxygen fraction to maintain oxygen saturation ≥96%. The respiratory rate was adjusted to maintain end-tidal CO_2_ between 35 and 40 mm Hg. An infusion of 3 mL·kg^−1^·h^−1^ of ringer lactate (RL) solution was started as fluid maintenance. 

### 2.1. Hemodynamic Management

In the GDT group, the anesthesiologists applied perioperative GDT according to the institutional optimization protocol. After induction of general anesthesia, the maximal value of stroke volume index (SVI) was defined as the absence of a sustained rise in SVI of at least 10% for more than 10 min in response to a fluid challenge. No more than 500 mL of ringer lactate solution was administered for the initial determination of the maximal value of SVI before the beginning of the surgical procedure. Further 250 mL fluid challenges were administered only when SVI decreased > 10% or when stroke volume variation (SVV) was >13%. SVI optimization was maintained during surgery, with subsequent boluses of fluids as required. At the same time, if the MAP was <65 mm Hg, vasoactive drugs were administered (see Supplemental Figure S1). 

In the HPI group, the HPI parameter was displayed on the HemoSphere screen in addition to other hemodynamic parameters. When the HPI reached a value of 85 or more, the number blinked red, and an audible alarm alerted the anesthesiologist to the risk of hypotension. In this eventuality, the HemoSphere monitor with the Acumen software displayed a secondary screen with the following additional variables: SVV, the peak rate of arterial pressure (dP/dt_max_) and the dynamic arterial elastance (Ea_dyn_, defined as PPV/SVV), providing information about the underlying cause of the impending hemodynamic instability. To standardize the interpretation of hemodynamic parameters, a therapeutical GDT “modified” protocol was established, considering the main mechanisms of hypotension (hypovolemia, vasoplegia and decreased contractility). Recommended potential interventions were fluids, fluids plus vasopressor, vasopressor or inotrope [22]. A ringer lactate solution volume of 250 mL was considered a fluid bolus. When required, vasopressor norepinephrine was started at a dose of 0.1 mcg·kg^−1^·min^−1^ and increased if necessary. If inotrope was needed, dobutamine was started at a dose of 2.5 mcg·kg^−1^·min^−1^ and increased if necessary (see Supplemental Figure S2).

After the surgery, the patients were transferred to the post-anesthesia care unit or to the ICU according to the clinician’s judgment.

Data were retrieved from electronic medical records of the hospital, and included the following: patients’ baseline characteristics, surgery type and duration, medical devices used during the procedure, medical procedures (e.g., diagnostics tests, imaging, consultancy, wound dressings, etc.), hospital length of stay (LOS), ICU admission and LOS. Hospitalization costs were estimated according to Table 1. 

### 2.2. Statistical Analysis 

Continuous variables are expressed as mean ± SD or median [25th–75th], while categorical variables are expressed as numbers (percentages). The normality of variables was assessed using the Shapiro–Wilk test. Continuous variables were compared with a two-sample *t*-test or Wilcoxon–Mann–Whitney test, based on the normality distribution. Categorical variables were evaluated using the chi-square test or Fisher’s exact test, as appropriate. A 2-sided probability value of *p* < 0.05 was considered statistically significant. 

A pre-specified subgroup analysis was conducted on the type of surgery, laparoscopic (LPS) versus laparotomic (LPT). 

An “a priori” sample size calculation was not performed, due to an absence of available economic data on HPI use.

## 3. Results

### 3.1. Patient’s Characteristics

Ninety-nine patients were retrospectively enrolled in the study, sixty-six patients in the control group and thirty-three in the HPI group, matched in a 2:1 ratio based on ASA physical status, diagnosis at admission, type of procedure, duration of surgery and age. The baseline characteristics and clinical outcomes are reported in Table 2. As shown, the matched variables were similar between the two groups. The patients’ population consisted of 40- to 70-year-old ASA II (80%) or III (20%) women undergoing laparoscopic (50%) or laparotomic (50%) surgery mainly for ovarian (54%) or endometrial cancer (33%). The rest (13%) were operated on for advanced cervical cancer or other procedures. 

### 3.2. Surgery and Hospitalization

The median surgical duration was about three and a half hours and the median hospital LOS was 4 days plus 1 day for the night before surgery. Seven patients in the control group and two patients in HPI group were admitted into the ICU postoperatively, with a total of 16 days in the control group and 6 days in the HPI group, without statistical differences between the groups. All patients were discharged with referral to rehabilitation according to the specific clinical pathway, apart from two patients in the control group; one was discharged to other healthcare facilities, and one died during the hospital stay. 

### 3.3. Cost Analysis

**Figure 1:**
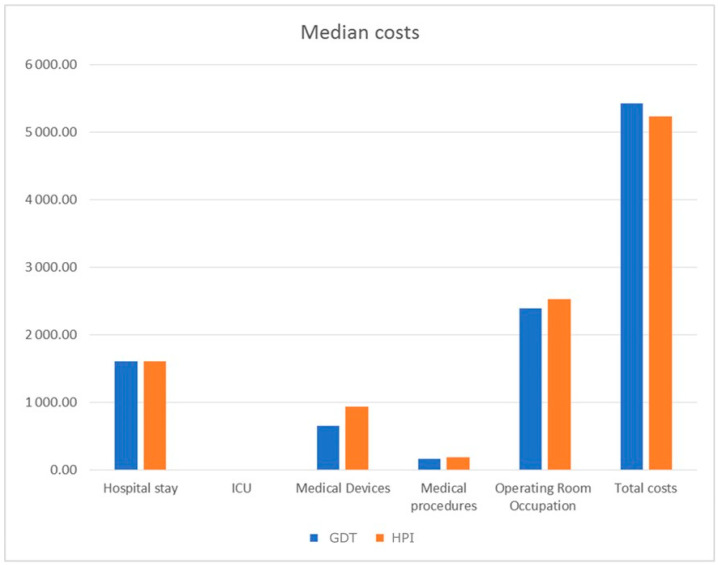
Median costs expressed in EUR of hospitalization and surgery in HPI (hypotension prediction index) and control GDT (goal-directed therapy) groups. ICU: intensive care unit.

The associated costs are presented in Figure 1. The only statistically significant difference was found in the field of medical devices, with higher costs in the HPI group, EUR 937 (764, 1514) versus EUR 650 (375, 1516) in the control group, *p* = 0.046. However, the median total cost of hospitalization and surgery was EUR 5425 (3505, 8127) in the control group and EUR 5227 (4201, 7023) in the HPI group (*p* = 0.697). Indeed, operating room occupation was the most relevant contributor, accounting for 44% of costs in the control group and 48% in the HPI group, followed by hospital stay costs (30% in both groups) and medical devices (12% in the control group vs. 18% in the HPI group). Results of the subgroup analysis are shown in Table 3. Similar numbers of patients in the control and HPI groups underwent laparoscopic and laparotomic surgery in equal proportions, with similar baseline characteristics and surgical duration, but also similar hospital LOS. However, in the laparotomic groups, a higher proportion of patients was diagnosed with ovarian cancer and, along with surgical complexity, the intraoperative time and postoperative LOS, including ICU days, were longer compared to the laparoscopic groups. Accordingly, the costs for laparotomic surgery were higher, more than EUR 8000 versus roughly EUR 4000 in the laparoscopic groups, but no differences were noted in the total costs between the HPI and control groups. Notably, while medical devices expenses were still statistically higher in the laparoscopic HPI group compared to control (EUR 794 (762, 1262) vs. EUR 445 (300, 833), *p* = 0.028), the greater expenditure in medical devices was not statistically significant for the laparotomic HPI group (EUR 1417 (792, 2504) vs. 1381 (EUR 557, 1827), *p* = 0.297). In addition, there was a tendency towards lower median total hospital costs in the laparotomic HPI group of about EUR 1000 compared to the laparotomic control group (EUR 7023 (5227, 11,438) vs. EUR 8005 (5961, 9679), *p* = 0.945), mainly supported by a decrease in hospital and ICU LOS, although non-statistically significant.

## 4. Discussion

The primary finding of this study is that the implementation of a GDT hemodynamic algorithm together with the Acumen IQ sensor technology in gynecologic oncologic surgery is associated with similar total costs, despite the medical device disposables. The application of HPI-based guidance does not lead to an increase in medical devices-associated costs, with a trend towards reduced total hospital costs, although not significant. This seems compatible with a scenario of cost neutrality, with the indication of possible benefits from the additional costs related to HPI-Acumen technology in settings at high risk of perioperative complications. 

The study did not clearly show a reduction in hospital LOS or ICU admission, although a downward trend (6 days HPI group, 5 days control group for hospital LOS) is present in the laparotomic HPI group. This analysis leads to the assumption that the ‘marginal gain’ of reducing the hospital LOS in the laparotomic HPI group of just one day can very well compensate for the increased medical device expenses, and avoiding ICU admission is associated with an even higher cost–benefit ratio. In fact, medical device disposables account for less than 20% of the total costs, while hospital stay equals about 30% of total costs.

A positive economic effect of the HPI software is more likely to be seen where patient’s physical status, comorbidities, surgical time, complexity and duration and intraoperative blood loss increase the burden of hypotension-related complications and, consequently, hospitalization costs. Indeed, laparotomic surgery is associated with increased complications and longer LOS [28], making this cohort especially relevant for reducing hospital costs. 

IOH is a common finding during gynecologic oncologic surgery [17,21,22,29,30]. The amount of hypotensive load varies between different reports based on the cancer severity, characteristics of the patient, anesthetic technique and institutional protocol for hemodynamic management [22,30,31]. Previous research clearly established the relationship between IOH and serious postoperative outcomes in non-cardiac surgery patients [2,3,4,5,6,7,8]. Given these findings, it is reasonable to consider the association of IOH with resource use: this is a transverse problem that involves physicians, hospital administrators, and government. Recent findings suggest that interventions for preventing IOH and postoperative functional impairment may plausibly be associated with significant cost savings [32].

The primary contribution of this research is the economic evaluation of both standardized hemodynamic management and personalized goal-directed pro-active management. It is very interesting to note that cost analysis of both personalized-preventive and standard strategies takes into account various risk groups, as shown by the different ASA scores of the patients enrolled.

This study has several limitations. First of all, conclusions about clinical outcomes and their consequences in management and treatment can only be drawn to a limited extent given the retrospective nature of this study. We acknowledge that case matching and stratification are not surrogates of a prospective randomized trial. Economic evaluations, therefore, have a diminished robustness. Secondly, IOH was not measured due to the retrospective nature of the study: a future prospective analysis that correlates the amount of IOH with economic costs could be interesting. Thirdly, the small number of cases, which was not set up in any previous sample size calculation based on clinical and economic outcomes, does not allow for a reliable statement regarding healthcare utilization endpoints such as ICU or hospital LOS. The cost comparison analysis in this study was conducted with a pragmatic approach, and retrospectively analyzed real-world data to obtain initial indicators for future solid study designs from this perspective, such as micro-cost analyses with calculated sample sizes, specific populations and predefined outcomes. In addition, a follow-up examination period of at least 30 days should be scheduled for an initial assessment of therapeutic benefits for the supplementary use of the HPI software and Acumen IQ sensor technology. Then, this information can also be used to determine effects in the outlay for follow-up examinations, which remains open in this study.

## 5. Conclusions

This retrospective cost evaluation in gynecological surgery has shown relevant indicators in the evaluating the benefits of complementary HPI software and Acumen IQ sensor technology usage in perioperative settings. Specifically, trends towards benefits in economic endpoints are evident in open surgical application, in particular with regard to the overall hospital costs in this group.

Robust cost–benefit assessments require advanced health economic evaluations with appropriate study designs.

## Figures and Tables

**Table 1:** Criteria to estimate hospital costs. The total operating room (OR) time was defined as the time between entry and exit of the patient into the OR. The surgical time was defined as the time between start and end of the surgery.

Costs	Criteria to Estimate Hospital Costs
Hospital stay	EUR 320/day
ICU	EUR 1200/day
Operating room	-anesthesiologist: EUR 60/h total OR time -1st and 2nd surgeon: EUR 60/h + 45/h surgical time -hospital room personnel and pharmaceutical products: EUR 6.5/min total OR time
Medical devices	Hospital’s real costs for medical devices involved in surgery
Medical procedures	The services/diagnostic tests are estimated at the value of the outpatient tariff or, if absent, at the internal value applied for consultancy

**Table 2:** Baseline characteristics and clinical outcomes of the population. Data are presented as mean ± standard deviation, median (25th percentile, 75th percentile) or counts (percentage).

	Control (*n* = 66)	HPI (*n* = 33)	*p*-Value
Age (years)	58 ± 11	59 ± 15	0.862
ASA Physical status, *n* (%)			0.904
I	2 (3)	1 (3)	
II	52 (79)	25 (76)	
III	12 (18)	7 (21)	
Admitting diagnosis			0.565
Cervical cancer	5 (8)	1 (3)	
Ovarian cancer	35 (53)	18 (55)	
Uterine cancer	24 (36)	12 (36)	
Endometrial hyperplasia	2 (3)	1 (3)	
Uterovaginal prolapse	0 (0)	1 (3)	
Year of surgery			<0.001
2020	39 (59)	33 (100)	
2021	27 (41)	0 (0)	
Type of surgery			>0.999
Laparoscopic	32 (48)	16 (48)	
Laparotomic	34 (52)	17 (52)	
Duration of surgery (minutes)	204 (152, 305)	209 (158. 288)	0.862
Hospital LOS before surgery (days)	1 (1, 1)	1 (1, 1)	0.232
Hospital LOS after surgery (days)	4 (2, 6)	4 (3, 5)	0.688
ICU LOS, total days	16	6	
ICU LOS (day/patient)	0 (0, 0)	0 (0, 0)	0.82

*p*-value refers to *t*-test, Mann–Whitney or chi-square test. HPI: hypotension prediction index, ASA: American Society of Anesthesiologists, LOS: length of stay, ICU: intensive care unit.

**Table 3:** Baseline characteristics, clinical outcomes and table of costs of subgroups undergoing laparoscopic or laparotomic surgery. Data are presented as mean ± standard deviation, median (25th percentile, 75th percentile) or counts (percentages).

	Laparoscopic Surgery			Laparotomic Surgery		
	Control (*n* = 32)	HPI (*n* = 16)	*p*-Value	Control (*n* = 34)	HPI (*n* = 17)	*p*-Value
Age (years)	57 ± 11	58 ± 12	>0.999	58 ± 11	60 ± 17	0.749
ASA Physical status, *n* (%)						
I	2 (6)	1 (6)	0.867	0 (0)	0 (0)	>0.999
II	24 (75)	11 (69)		28 (82)	14 (82)	
III	6 (19)	4 (25)		6 (18)	3 (18)	
Admitting diagnosis			0.654			0.907
Cervical cancer	1 (3)	0 (0)		4 (12)	1 (6)	
Ovarian cancer	14 (44)	8 (50)		24 (71)	12 (71)	
Uterine cancer	15 (47)	6 (38)		6 (18)	4 (24)	
Endometrial hyperplasia	2 (6)	1 (6)		0 (0)	0 (0)	
Uterovaginal prolapse	0 (0)	1 (6)		0 (0)	0 (0)	
Year of surgery						
2020	18 (56)	16 (100)	0,002	21 (62)	17 (100)	0.002
2021	14 (54)	0 (0)		13 (38)	0 (0)	
Duration of surgery (minutes)	157 (131, 183)	152 (111, 190)	>0.999	302 (229, 356)	283 (231, 377)	0.842
Duration of surgery (minutes)						
Hospital LOS before surgery (days)	1 (1, 1)	1 (1, 1)	0.516	1 (1, 1)	1 (1, 1)	0.425
Hospital LOS after surgery (days)	2 (2, 3)	3 (2, 3)	0.693	6 (5, 9)	5 (5, 7)	0.206
ICU LOS, total days	1	0		15	6	
ICU LOS (day/patient)	0 (0, 0)	0 (0, 0)	0.494	0 (0, 0)	0 (0, 0)	>0.999
Costs in euro						
Hospital stay	960 (640, 1600)	1280 (960, 1360)	0.376	2240 (1600, 3120)	1920 (1600, 2560)	0.314
ICU stay	0 (0, 0)	0 (0, 0)	0.494	0 (0, 0)	0 (0, 0)	>0.999
Operating room occupation	2042 (1581, 2211)	1757 (1235, 2287)	0.741	3510 (2604, 4411)	3405 (2777, 4012)	0.929
Medical devices	445 (300, 833)	794 (762, 1262)	0.028	1381 (557, 1827)	1417 (792, 2504)	0.297
Medical procedures	128 (94, 184)	154 (116, 214)	0.4	217 (152, 352)	204 (105, 264)	0.4
Total costs	3618 (3073, 4406)	3955 (3618, 5103)	0.379	8005 (5961, 9679)	7023 (5227, 11,438)	0.945

*p*-value refers to *t*-test, Mann–Whitney or chi-square test. HPI: hypotension prediction index, ASA: American Society of Anesthesiologists, LOS: length of stay, ICU: intensive care unit.

## Data Availability

All data may be shared under reasonable request.

## Supplementary Materials

The following supporting information can be downloaded at: https://www.mdpi.com/article/10.3390/jpm14010058/s1, Figure S1: Treatment algorithm for the GDT group; Figure S2: Treatment algorithm for the HPI group.
