# Supplementary material for: Personalized Predictive Hemodynamic Management for Gynecologic Oncologic Surgery: Feasibility of Cost–Benefit Derivatives of Digital Medical Devices
Source: J Pers Med. 2023 Dec 30;14(1):58. doi: 10.3390/jpm14010058 (PMC10820080; doi:10.3390/jpm14010058)

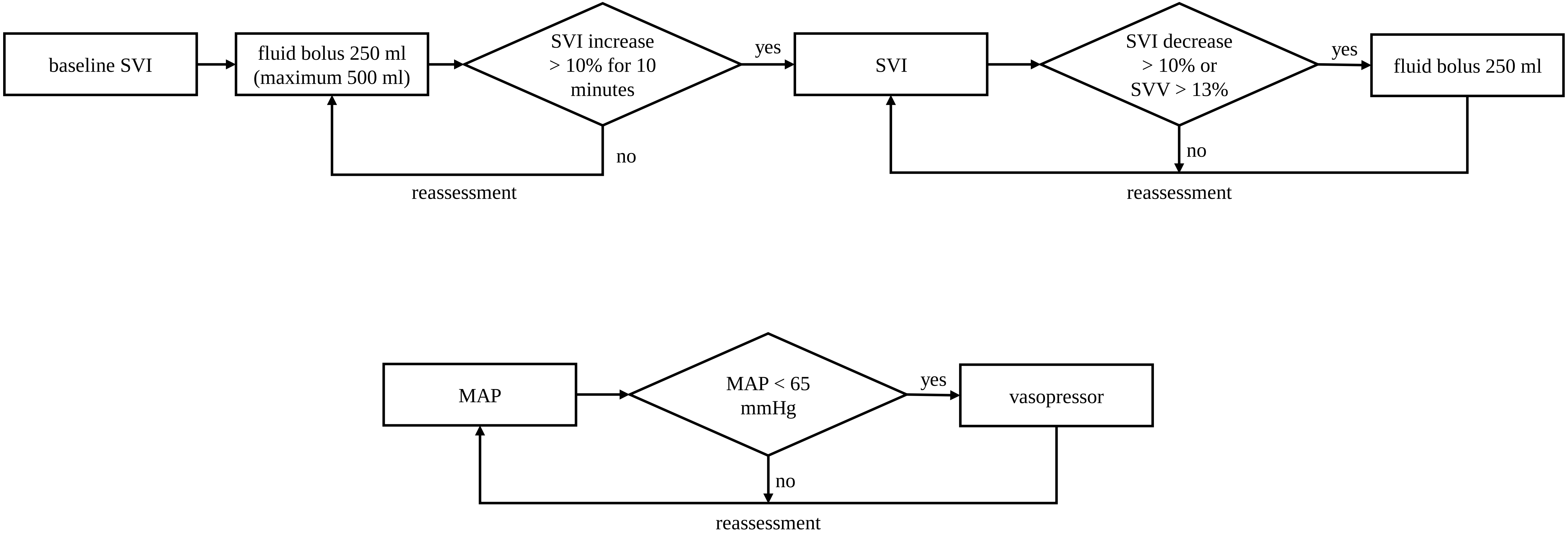


**Supplemental Figure 1**. Treatment algorithm for the GDT group.
MAP: mean arterial pressure. SVV: stroke volume variation. SVI: stroke volume index.

**Supplemental Figure 2**. Treatment algorithm for the HPI group.
HPI: Hypotension Prediction Index. MAP: mean arterial pressure. SVV: stroke volume variation. $dP/dT_{max}$: peak rate of arterial pressure. $Ea_{dyn}$: dynamic arterial elastance SVI: stroke volume index.

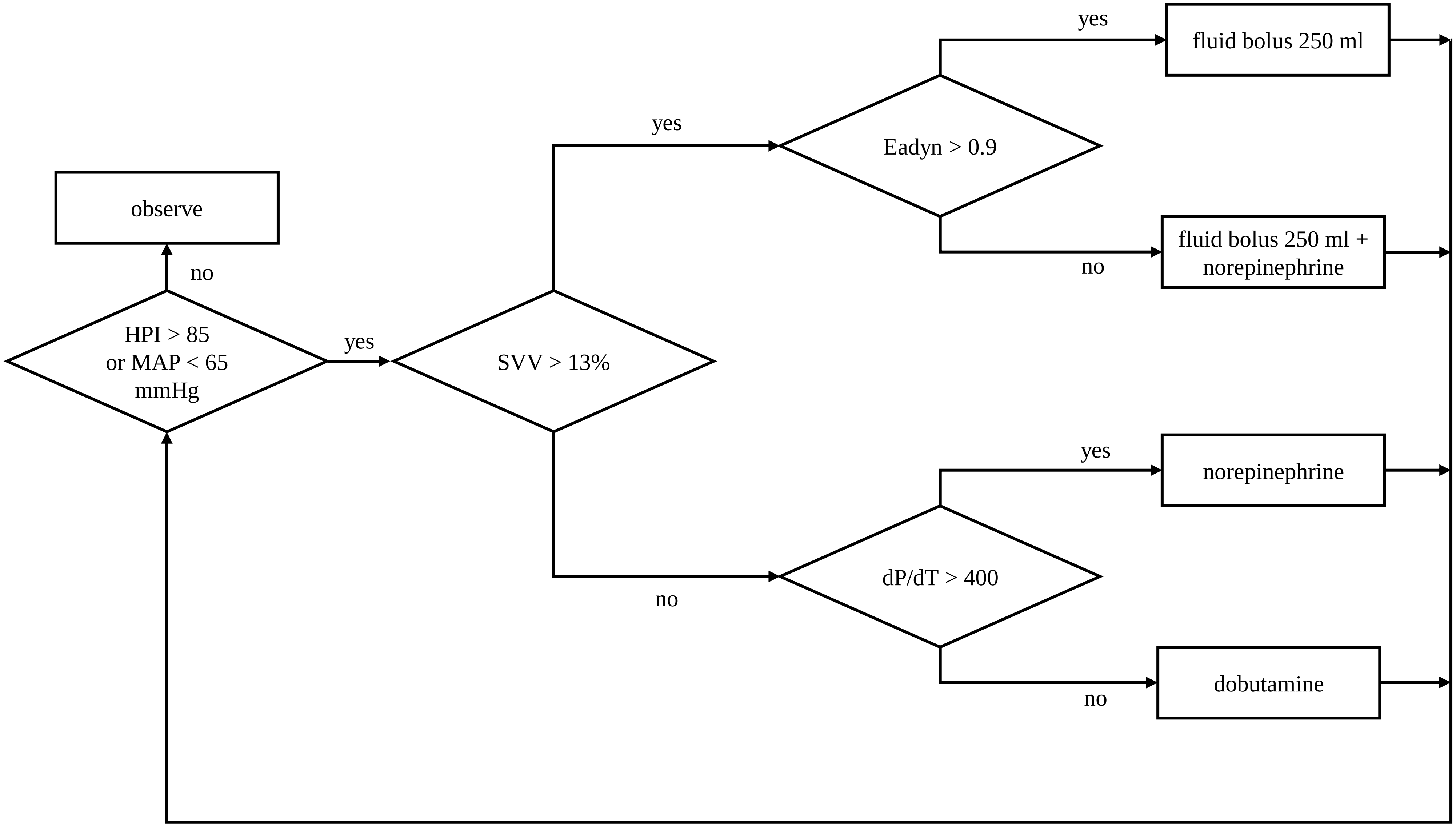

Supplement: Supplementary file 1 [file jpm-14-00058-s001.zip › jpm-2760632-supplementary.pdf]
